# Quality of Life Related to Visual Function in Three Young Adults with Mucopolysaccharidoses

**DOI:** 10.1100/tsw.2003.88

**Published:** 2003-10-05

**Authors:** Katherine L. Bergwerk, Yaron S. Rabinowitz, Rena L. Falk

**Affiliations:** Cornea-Genetic Eye Institute, Cedars-Sinai Medical Center, Los Angeles, CA, USA

**Keywords:** mucopolysaccharidoses, cornea, vision, quality of Life, QOL, human development, United States

## Abstract

The systemic mucopolysaccharidoses are complex syndromes, which may include corneal clouding as a mechanism leading to decreased vision and hence decreased quality of life. This study presents three young adult patients with mucopolysaccharidoses in order to compare their visual status through retrospective chart review, including patient and guardian interview, history, and examination, including a modified form of the VF-14 questionnaire (visual function with 14 questions).When the visual acuity and VF-14 results of the three patients were compared, the results of the VF-14 correlated with the patients' visual acuity status. The two patients who retained clear corneas or underwent penetrating keratoplasty had a wider range of social and physical activities, and an overall better quality of life than did the patient with decreased vision due to opacified corneas.We conclude that close monitoring of the ocular health of patients with storage syndromes that may compromise visual acuity must be stressed, and intervention to insure good vision is of the utmost importance to maintaining a good quality of life for these patients, especially as new therapies assist these patients to achieve increased longevity with better health.

## INTRODUCTION

Corneal clouding is a prominent feature in many of the systemic mucopolysaccharide storage disorders (MPS), including Maroteaux-Lamy syndrome (MPS VI) and Sly disease (MPS VII)[1,2]. The resulting diminished vision may lead to a decrease in the patient's level of function and quality of life. Various methods of therapy are currently under exploration to treat the protean manifestations of mucopolysaccharidoses, including enzyme replacement, gene therapy, and bone marrow transplantation[3]. Penetrating keratoplasty has been used to treat the corneal manifestations[4]. Here we present three patients with mucopolysaccharidoses, who have survived into their third decade of life and compare the relationship of their visual acuity to their activities of daily living, as a measure of their quality of life. The VF-14 is an acknowledged standard tool to assess subjective visual function. It was first used to assess the impact of cataract on patient life and has since been used for other diseases, such as glaucoma, myopia, and macular degeneration[5]. A modified form of the widely utilized VF-14 test instrument was prepared in our office that is better suited for this patient population (see Appendix). Patients with the severe form of MPS VI may survive until the second or third decades of life until cardiopulmonary complications ensue, while MPS VII is similarly associated with survival into adolescence or adulthood in the milder forms. As we are better able to manage the other systemic problems manifested by these patients, continued attention must be paid to their ophthalmic complications such as corneal clouding, glaucoma, and optic nerve compression due to deposition within the optic nerves, or from hydrocephalus.

## METHODS

Retrospective chart review of patient charts which included interview information, review of systems, as well as physical examinations. Visual function was assessed in part by using a modified form of the standard index VF-14 questionnaire, which assesses subjective visual function.

## RESULTS

A comparison of the three patients is presented in Table 1. For comparison, Figure 1 shows the corneas of the patients involved. Figures 1a and 1b demonstrate the difference between patient 1 pre- and postcorneal transplant surgery. Figure 1c demonstrates patient 2 with mild peripheral opacities, which do not significantly alter the patient's vision. Figure 1d demonstrates the dense thick opaque corneas of patient 3.

Patient 2 has the best visual acuity and the best score on the modified visual function assessment. Patient 1 has vision in the low vision range and has a reduced score on the visual assessment questionnaire. Patient 3, although she has the least mental impairment, has the worst visual impairment, and the worst score on the visual function form. The results of the patients' visual function assessment correlated with the visual acuity and with the clarity of the patients' degree of corneal clarity.

## DISCUSSION

MPS VI (Maroteaux-Lamy) has a wide range of clinical severity, including a milder and a more severe phenotype. Patient 3 has the more severe form, with symptoms such as corneal clouding present in early childhood. While corneal clouding progressed slowly, the patient also developed optic nerve atrophy from optic canal compression, which was relieved by ventriculo-peritoneal shunt placement at age 7. She regained some vision thereafter and was treated briefly for glaucoma. Scleral thickening was evident on ultrasound imaging studies. At age 20 years, an electro-retinogram was performed and demonstrated abnormal rod function with normal cone function. The test results were consistent with visual acuity better than the patient's current level of bare light perception, without color or directionality perception. This led to the conclusion that the patient's vision could be significantly improved by penetrating keratoplasty to remove the thick corneal haze.

**TABLE 1**

**Comparison of Patients**

| | Patient 1 | Patient 2 | Patient 3 |
|---|---|---|---|
| **MPS** | VII | VII | VI |
| **Gender** | M | M | F |
| **Current age** | 22 | 27 | 22 |
| **Enzyme level** | 0.9 nmol/h/ml | 1.0 nmol/h/ml | N/A |
| **Mental retardation** | Mild-moderate | Mild-moderate | None |
| **Systemic manifestations** | Tracheostomy | Attenuated/ruptured bowel | Seizures |
| | Cardiac (MR) | Cardiac (AI) | Cardiac |
| | Scoliosis | Scoliosis | Contractures |
| | Spinal decompression | Spinal fusion | Hydrocephalus/VP shunt |
| | Hand contractures | Hernias | Hernias |
| | Lymphadenopathy | Seasonal allergies | |
| | Multiple caries | Multiple caries | |
| **Ocular history** | Corneal transplants | Mild hyperopia /astigmatism | Opaque/edematous corneas |
| | OD-1996 | Faint corneal opacities | Glaucoma |
| | OS-1998 | | Optic atrophy |
| | | | Exophthalmos |
| **Current visual acuity** | OD: 20/100 | OD: 20/60 | OD: Light perception |
| | OS: 20/200 | OS: 20/70 | OS: Light perception |
| | High school/special | Special education workshop | None since 1985 |
| **School** | Vision therapy program | | |
| **Social activities** | Job at store | Church classes | None (homebound) |
| | Home visitors | Siblings’ friends | |
| **Hobbies** | Recycling, games, TV | Playing ball, computer, camping | Listens to radio/TV |
| | | Family trips, games | |
| **Visual function score** | 8 | 1 | 33 |

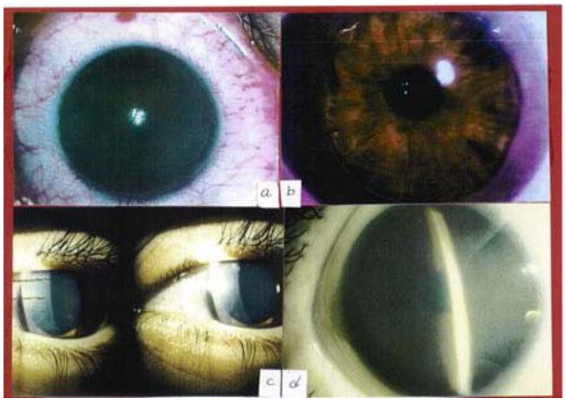

**FIGURE 1.** Comparison of corneal opacities: (a) patient 1 prior to surgery with opaque, thick cornea; (b) patient 1 with clear graft following corneal transplant; (c) patient 2 with mild, peripheral stromal opacities; (d) patient 3 with dense, thick, opaque cornea.

As with most MPS VI patients, she had no evidence of mental retardation. However, she suffered from depression secondary to her degree of debilitation. She has refused corneal transplantation, not due to fear of the procedure, but because she refuses medical examinations. After tenth grade, she left school. She received home schooling for 1 year and then refused the teacher's visits. She does not leave her home, except for emergency hospital visits, and spends her waking hours listening to the radio, television, or audio-books. She is not engaged in any social activities outside the home, and has no visitors. On our assessment of visual function, her score was 33, in which the best score equals 0 and the worst equals 36. In contrast, patient 1 scored 8 and patient 2 scored 1.

The two male patients with MPS VII had many contrasts to the above patient. Both have mild-moderate mental retardation, which would seem to limit their interactions more than those of the patient with normal intelligence. However, both have adequate vision to enable them to pursue their activities of daily living and to engage in social activity. Patient 1 attends a school with a special vision stimulation program. He can manipulate his wheelchair to go around the neighborhood independently, and does so to collect cans, which he recycles to collect money. He has a job at a local store reshelving misplaced items. He likes to play ball and has a friend who comes to visit. He enjoys the company of others, especially his vision therapist. The second MPS VII patient, who did not require any intervention to maintain his vision, is able to travel with his family, attends religious school classes, and was able to receive communion and take his religious vows. He enjoys the social interaction with his weekly religious school classmates, and attended a workshop with art and computer activities.

## CONCLUSION

Mucopolysaccharidoses types VI and VII are progressive, debilitating diseases with manifestations affecting many organ systems. Corneal clouding leading to visual compromise is a prominent feature of these disorders. Here we demonstrate, albeit with a small group of patients, that appropriate visual care with an ophthalmologist, including regular examinations, may aid in early diagnosis of complications and thereby allow early and aggressive intervention such as treatment of glaucoma or optic nerve compression. If needed, surgical intervention such as penetrating keratoplasty may be highly beneficial to maintaining good vision, hence improving the patient's overall quality of life. In addition to other modes of therapy, e.g., bone marrow or stem cell transplantation that may or may not have an effect on the cornea, corneal transplantation is an important consideration, despite existing ocular comorbidities or limited life expectancy.

## ACKNOWLEDGMENTS

This study was supported in part by NIH NEI EY00418 to K. Bergwerk and NIH grant to Y. Rabinowitz.

## BIOSKETCHES

**Katherine L. Bergwerk, M.D.,** is an ophthalmic geneticist at Cedars-Sinai Medical Center in Los Angeles and an Associate Clinical Professor of Ophthalmology at the Jules Stein Eye Insttitute, UCLA. Her clinical research interests are primarily focused on ocular problems in Down syndrome, as well as ocular manifestations of inherited metabolic diseases. E-mail: Bergwerk@juno.com

**Yaron S. Rabinowitz, M.D.,** is the Director of Ophthalmology Research at Cedars-Sinai Medical Center and a Clinical Professor of Ophthalmology at the Jules Stein Eye Institute, UCLA School of Medicine. Website: http://www.laser-prk.com

**Rena E. Falk, M.D.,** is Director of the Prenatal Diagnosis Center and Medical Director of the Cytogenetics Laboratory at Cedars-Sinai Medical Center in Los Angeles. She is also a Professor of Pediatrics at UCLA School of Medicine who has published numerous articles, chapters, and abstracts relating to clinical genetic and cytogenetic conditions. She has particular interest in the genetic aspects of hearing loss and deafness, in delineation of clinical syndromes, and in genotype to phenotype correlations in Mendelian and cytogenetic disorders. E-mail: Rena.Falk@cshs.org

## APPENDIX

Modified form of VF-14 (visual function with 14 questions) assessment geared towards patients with mental retardation/developmental disability/intellectual disability.

Name of patient:

Date:

**1. Does the patient have any difficulty identifying product labels, e.g., cereal boxes, soda cans?**

Yes/No/Not applicable

If yes, how much difficulty does the patient currently have?

1. A little
2. A moderate amount
3. A great deal
4. Unable to do the activity

Was the patient previously able to perform this task? If so, when?

**2. Does the patient have any difficulty, even with glasses, recognizing people when they are close to him/her?**

Yes/No/Not applicable

If yes, how much difficulty does the patient currently have?

1. A little
2. A moderate amount
3. A great deal
4. Unable to do the activity

Was the patient previously able to perform this task? If so, when?

**3. Does the patient have any difficulty, even with glasses, seeing steps, stairs, and curbs?**

Yes/No/Not applicable

If yes, how much difficulty does the patient currently have?

1. A little
2. A moderate amount
3. A great deal
4. Unable to do the activity

Was the patient previously able to perform this task? If so, when?

**4. Does the patient have any difficulty, even with glasses, seeing traffic signs, street signs, or store signs?**

Yes/No/Not applicable

If yes, how much difficulty does the patient currently have?

1. A little
2. A moderate amount
3. A great deal
4. Unable to do the activity

Was the patient previously able to perform this task? If so, when?

**5. Does the patient have any difficulty, even with glasses, doing handicrafts, such as coloring or using scissors or glue?**

Yes/No/Not applicable

If yes, how much difficulty does the patient currently have?

1. A little
2. A moderate amount
3. A great deal
4. Unable to do the activity

Was the patient previously able to perform this task? If so, when?

**6. Does the patient have any difficulty, even with glasses, taking part in sports activities, such as catching or throwing a ball?**

Yes/No/Not applicable

If yes, how much difficulty does the patient currently have?

1. A little
2. A moderate amount
3. A great deal
4. Unable to do the activity

Was the patient previously able to perform this task? If so, when?

**7. Does the patient have any difficulty, even with glasses, playing games such as cards?**

Yes/No/Not applicable

If yes, how much difficulty does the patient currently have?

1. A little
2. A moderate amount
3. A great deal
4. Unable to do the activity

Was the patient previously able to perform this task? If so, when?

**8. Does the patient have any difficulty, even with glasses, eating independently?**

Yes/No/Not applicable

If yes, how much difficulty does the patient currently have?

1. A little
2. A moderate amount
3. A great deal
4. Unable to do the activity

Was the patient previously able to perform this task? If so, when?

**9. Does the patient have any difficulty, even with glasses, watching television?**

Yes/No/Not applicable

If yes, how much difficulty does the patient currently have?

1. A little
2. A moderate amount
3. A great deal
4. Unable to do the activity

Was the patient previously able to perform this task? If so, when?

At what distance does the patient sit from the television?

**Score: ___________/36**
